# Nanodiamond Theranostic for Light-Controlled Intracellular Heating and Nanoscale Temperature Sensing

**DOI:** 10.1021/acs.nanolett.1c00043

**Published:** 2021-04-21

**Authors:** Yingke Wu, Md Noor A Alam, Priyadharshini Balasubramanian, Anna Ermakova, Stephan Fischer, Holger Barth, Manfred Wagner, Marco Raabe, Fedor Jelezko, Tanja Weil

**Affiliations:** † Max Planck Institute for Polymer Research, Ackermannweg 10, 55128 Mainz, Germany; ‡ Institute of Inorganic Chemistry I, Ulm University, Albert-Einstein-Allee 11, 89081 Ulm, Germany; § Institute for Quantum Optics, Ulm University, Albert-Einstein-Allee 11, 89081 Ulm, Germany; ∥ Institute for Physics, Johannes Gutenberg University Mainz, Staudingerweg 7, 55128 Mainz, Germany; ⊥ Institute of Pharmacology and Toxicology, University of Ulm Medical Center, 89081 Ulm, Germany

**Keywords:** nanodiamond, nanogel, intracellular temperature manipulation and sensing, photothermal application

## Abstract

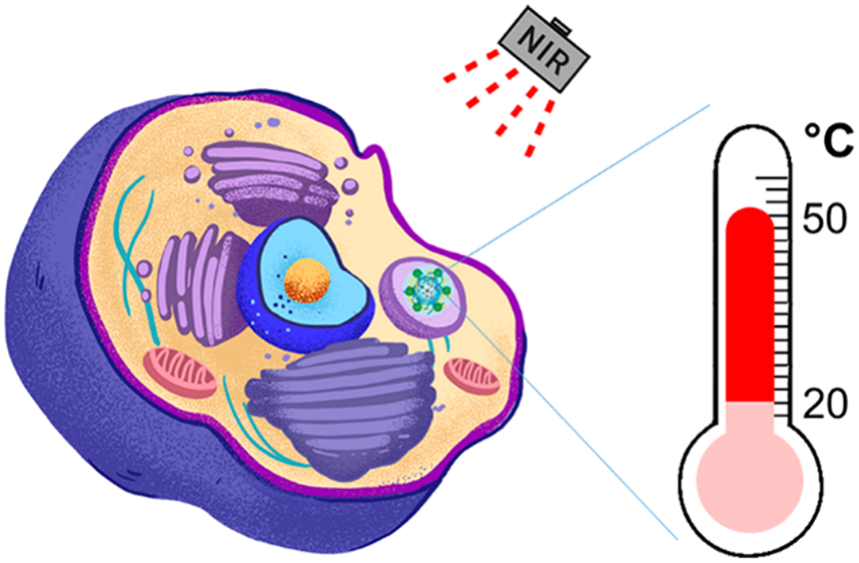

Temperature is an essential parameter in all biological systems, but information about the actual temperature in living cells is limited. Especially, in photothermal therapy, local intracellular temperature changes induce cell death but the local temperature gradients are not known. Highly sensitive nanothermometers would be required to measure and report local temperature changes independent of the intracellular environment, including pH or ions. Fluorescent nanodiamonds (ND) enable temperature sensing at the nanoscale independent of external conditions. Herein, we prepare ND nanothermometers coated with a nanogel shell and the photothermal agent indocyanine green serves as a heat generator and sensor. Upon irradiation, programmed cell death was induced in cancer cells with high spatial control. In parallel, the increase in local temperature was recorded by the ND nanothermometers. This approach represents a great step forward to record local temperature changes in different cellular environments inside cells and correlate these with thermal biology.

## Introduction

Temperature plays a fundamental role in biological processes of living organisms and is involved in cell differentiation, proliferation, and death,^1^ protein functions,^2,3^ and gene expression.^4^ Hence, probing or even manipulating the local intracellular temperature with high spatial control is crucial to understand the fundamental relationship between biological activities and temperature. Cells are very sensitive to temperature changes, which is exploited in photothermal therapy (PTT), where tumor cells^5,6^ or even the entire tumors^7−9^ are eliminated or shrink if the intracellular temperature exceeds 42 °C or even 50 °C, respectively. Counterintuitively, cell organelles such as mitochondria maintain a physiological temperature of close to 50 °C without harming the cell.^10^ Therefore, there is a fundamental interest in obtaining a deeper understanding of the local intracellular temperature as well as temperature changes during therapy and the effect on biological systems.

PTT is routinely used in patients to increase the local temperature in diseased cells or tissues, and it is effective in the treatment of certain cancers.^5,11^ During PTT, a photothermal agent (PA) is delivered into the tumor tissue and, upon illumination, the PA converts absorbed light energy into heat. Over time, this process leads to either partial or complete ablation of the tumor tissue.^12^ Various PAs have been designed, mainly in the form of nanomaterials, which benefit from the enhanced permeability and retention (EPR) effect of tumor tissue after an intravenous injection.^13,14^ However, these PAs lack the ability to sense and report local temperature changes, and it is not possible to correlate the induced local temperature and the effect on apoptosis. To improve the efficacy of PTT on the basis of rational information, the relationship between local temperature changes and induced controlled cell death via apoptosis would be crucial. Many luminescence-based thermometers have been reported that are combined with photothermal agents to monitor macroscopic temperature changes during PTT, such as lanthanide-doped nanoparticles^15,16^ and quantum dots.^17^ Different kinds of fluorescence-based thermometers have been explored to directly measure the intracellular temperature at the nanoscale;^18−20^ however, environmental parameters, such as pH, ion concentrations, and intracellular viscosity, could affect their sensitivity, resulting in an inaccurate temperature readout.^21^ Furthermore, the accuracy of nanoscale sensing in cells is still debated due to differences in the experimental readouts and calculated temperatures and thus developing new and sensitive temperature sensors that are not affected by environmental changes in their direct surroundings such as pH and solvent polarity will be valuable for many applications.^22−25^ Nanodiamonds (NDs) containing negatively charged nitrogen-vacancy (NV^–^) centers show neither photoblinking nor photobleaching,^26^ and their fluorescence is hardly influenced by pH, ion concentration, viscosity, molecular interactions, or organic solvents.^27^ The temperature response of NDs differs strongly from that of most fluorescence probes, as the NV^–^ center reveals a thermal shift of the zero-field splitting (*D*_0_) at 2.87 GHz (*m*_s_ = 0 to *m*_s_ = ±1)^28−30^ or the zero-phonon line (ZPL) at 637 nm.^31,32^ Thus, NDs could serve as highly sensitive nanoscale thermometers in biological systems.

Herein, we report the preparation and application of an intracellular local heat generator and nanothermometer, termed nanodiamond-nanogel-indocyanine green (ND-NG-ICG), serving as an intracellular self-reporting photothermal system to probe the local temperature changes during PTT. Upon irradiation, programmed cell death (apoptosis) was induced in cancer cells with high spatial control. In parallel, the local temperature increase was recorded inside endosomal vesicles of cancer cells. Unlike lanthanide-doped nanoparticles, the self-reporting ICG-coated ND system does not depend on the fluorescence intensity for temperature sensing and is therefore less affected by environmental changes such as pH and ionic strength. Our approach paves the way to ultimately optimize PTT on the basis of quantitative information. Moreover, we see great prospects to explore the effect of intracellular temperature changes on cellular processes in thermal biology.

## Preparation and Characterization of ND-NG-ICG

**Figure 1:**
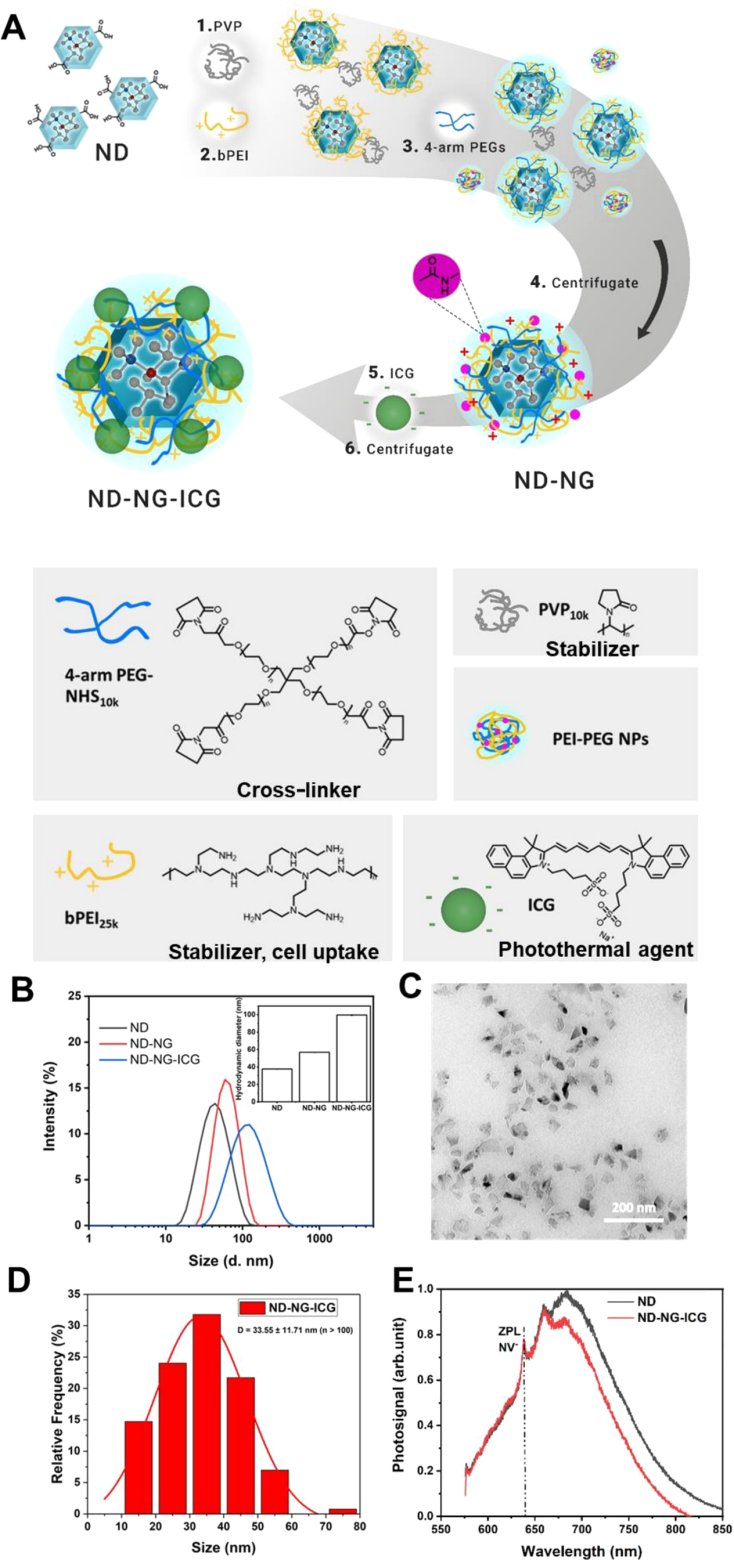
(A) Schematic illustration of the synthesis route of ND-NG-ICG. (B) Hydrodynamic diameters of ND, ND-NG, and ND-NG-ICG measured by DLS. (C) TEM image of ND-NG-ICG (scale bar 200 nm). (D) Histogram analysis of ND-NG-ICG (*n* > 100). (E) Normalized emission spectra (excitation at 532 nm) of ND and ND-NG-ICG (normalized to the maximum intensity of ND at 684 nm in the vibrational sideband). The zero-phonon line (ZPL) of NV^–^ is visible in both spectra.

NDs need to be stabilized by a biocompatible surface coating to prevent aggregation in biological media and to introduce reactive groups on the surface for postmodification. We have recently reported the stabilization of NDs in a cross-linked nanogel (ND-NG) that were nontoxic in cell experiments.^33^ Hyperbranched polyethylenimine (PEI) was used to precoat NDs in the presence of polyvinylpyrrolidone (PVP) as a stabilizer, and subsequently, a four-arm polyethylene glycol *N*-hydroxysuccinimide (NHS) ester was added to cross-link the precoated PEI on the surface of NDs in phosphate-buffered saline (PBS) buffer to form a stable, positively charged nanogel shell. Herein, we have adsorbed the anionic indocyanine green (ICG) by electrostatic interactions to yield ND-NG-ICG. Free ICG was removed from ND-NG-ICG by centrifugation (12000 rpm, Figure 1A). The amount of ICG that was loaded onto the ND-NG has a great effect on the efficiency of PTT. Therefore, the concentration of free ICG in the supernatant after centrifugation was quantified by measuring its characteristic absorbance at 789 nm. In this way, we avoid any interference of this method by the emission of the ND-NGs. 64.5 μg of ICG was loaded onto 200 μg of ND-NG, which corresponds to a loading efficiency of 24.4 wt % (Figure S1). In order to assess potential ICG leakage, the release of ICG was detected over time. In this case, ND-NG-ICG remained stable and only about 10% of ICG was released over 20 days (Figure S2). Since the hydrodynamic radius of nanoparticles greatly affects their cellular uptake, dynamic light scattering (DLS; Figure 1B) and transmission electron microscopy (TEM; Figure 1C,D) were performed to investigate the size distribution, shape, and morphology of ND-NG-ICG. In Milli-Q water, the hydrodynamic diameters of ND, ND-NG, and ND-NG-ICG increased from 37.7 ± 0.23 to 56.9 ± 0.27 and 99.6 ± 0.53 nm, respectively (Figure 1B). ND-NG-ICG revealed a monomodal size distribution (Figure 1B) with a polydispersity index (PDI) of 0.243, and no aggregates were observed in solution. In addition, the TEM image of the ND-NG-ICG analysis showed individual nanoparticles without any obvious aggregation (Figure 1C).

The photophysical properties of NDs containing NV^–^ centers are essential for the application of ND-NG-ICG in bioimaging and intracellular temperature sensing. The NV^–^ centers in NDs are very sensitive to the surface charge, and they can switch to the dark state (neutral NV center, NV^0^; positively charged NV center, NV^+^) under certain conditions. Importantly, in the dark state, they can no longer be used for sensing. To further study the influence of the polymer coating on the NV^–^ centers in NDs, ND-NG-ICG was dropped on a glass coverslip and spectra were recorded on a custom-built confocal microscope using 532 nm excitation with a power of 110 μW in front of the objective (oil, NA = 1.35). Upon irradiation of ND-NG-ICG at 532 nm, the emission spectrum of ND-NG-ICG revealed a slight decrease in the intensity of the peak at 680 nm due to a minor energy transfer component from ND to ICG because of the partial overlap of the emission spectra of ND and the absorption spectra of ICG (Figure S3). The zero-phonon line of NV^–^ at 637 nm was clearly visible without any background noise (Figure 1E), indicating that the NV^–^ centers in ND-NG-ICG remained in the optically active state, which is necessary for intracellular temperature sensing.

## ND-NG-ICG Reveals Cell Compatibility and Intracellular Localization Inside Endosomes

**Figure 2:**
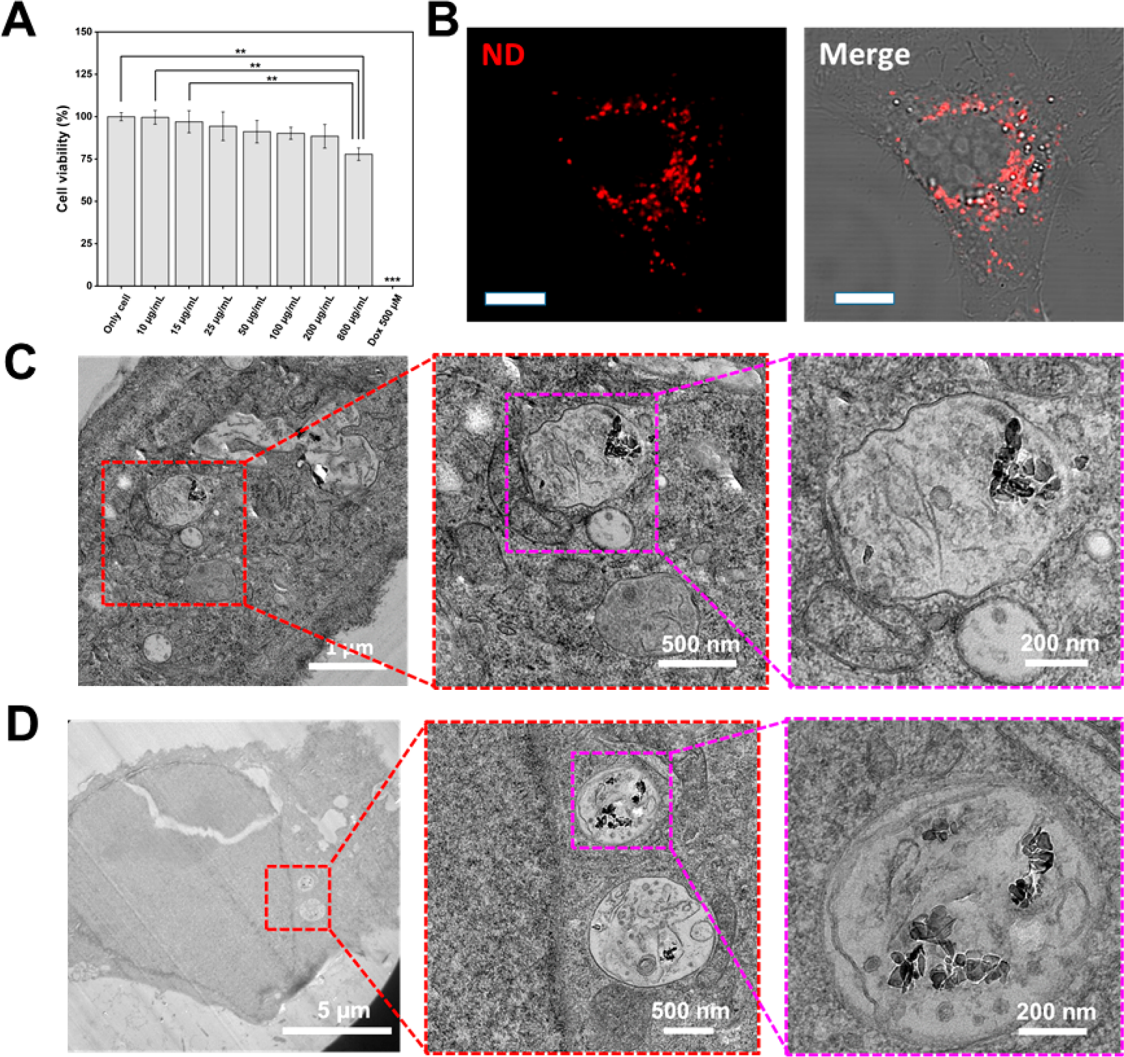
(A) Cell viability of HeLa cells after 24 h incubation with ND-NG-ICG (*n* = 3; one-way ANOVA, ** *p* < 0.01, *** *p* < 0.001). All other ND-NG-ICG concentrations are not statistically significant. Doxorubicin (Dox) was used as a negative control. (B) Confocal microscopy images of ND-NG-ICGs that were taken up into HeLa cells at a concentration of 100 μg/mL after 4 h (scale bar 10 μm). (C) TEM images of ND-NG-ICGs that were taken up into HeLa cells at a concentration of 10 μg/mL after 4 h. (D) TEM images of ND-NG-ICGs that were taken up into HeLa cells at a concentration of 100 μg/mL after 4 h.

The cell compatibility of ND-NG-ICG was investigated in a human cervical carcinoma cell line (HeLa). As displayed in Figure 2A, ND-NG-ICG showed high cell viability even after cell treatment using very high concentrations of up to 800 μg/mL. In addition, ND-NG-ICG was efficiently taken up by HeLa cells (Figure 2B), which did not alter the cell growth or the cell morphology. The intracellular localization of ND-NG-ICG was imaged after incubating HeLa cells for 4 h with two different nanoparticle concentrations, 10 and 100 μg/mL (Figure S10). Afterward, cells were processed for TEM measurement (see details in Methods in the Supporting Information and Figure 2C,D). We found internalized ND-NG-ICGs that were encapsulated in endosomal vesicles independent of the applied nanoparticle concentration, indicating no significant difference in the cell uptake mechanism between 10 and 100 μg/mL of ND-NG-ICG. At lower concentration (10 μg/mL ND-NG-ICG), fewer ND-NG-ICGs were found in cells.

## ND-NG-ICG-Based Temperature Sensing at the Nanoscale

**Figure 3:**
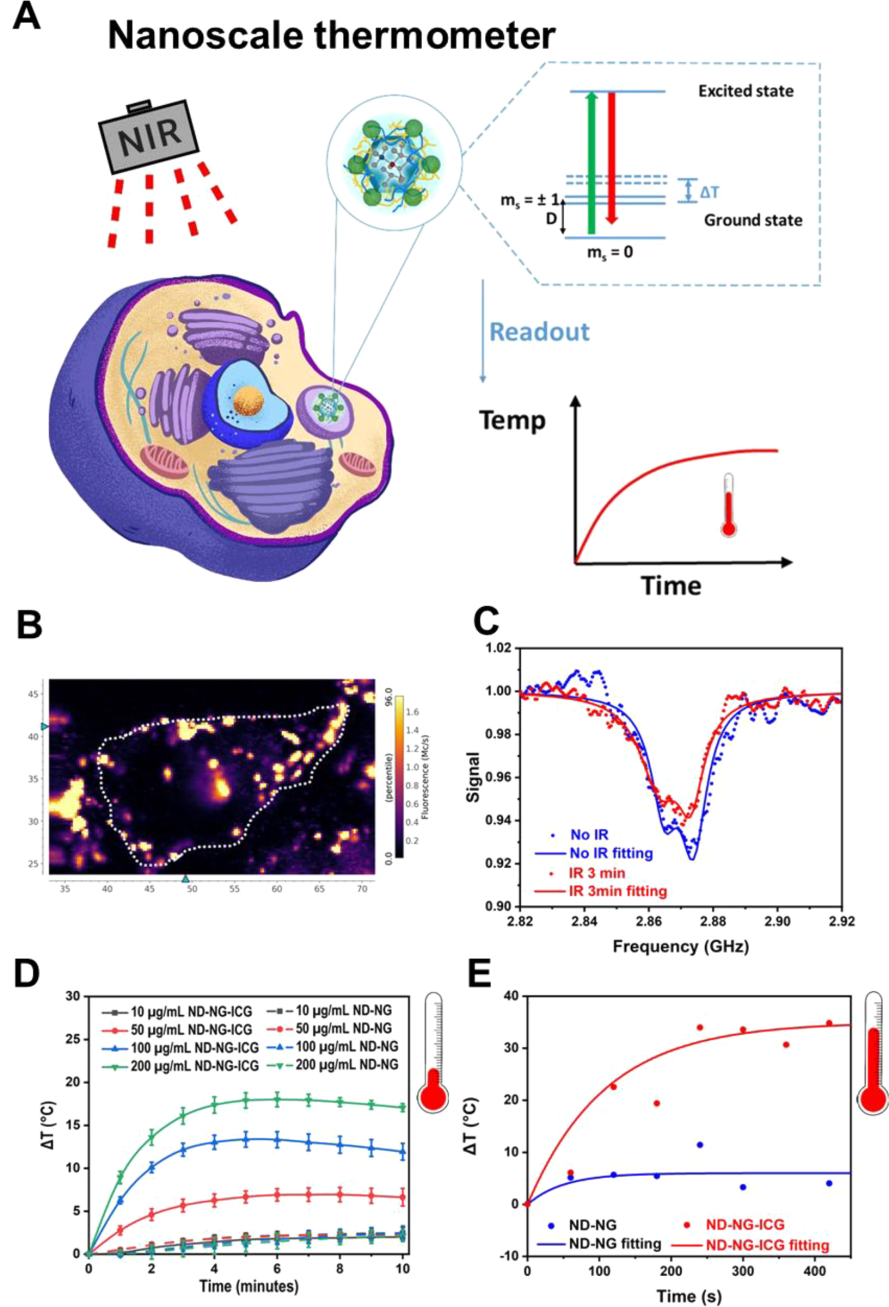
(A) Schematic presentation of the temperature measurements in living cells. Simplified energy level diagram of the NV^–^ center in ND displaying the ground state of the spin triplet and the excited state. Under zero magnetic field, the *m*_s_ = ±1 sublevels are split from the *m*_s_ = 0 state by the temperature-dependent zero-field splitting *D*_0_. The intracellular change in temperature can be monitored by the shift of the zero-field splitting parameter *D*_0_ using ODMR spectroscopy. At room temperature, *D*_0_ ≈ 2.87 GHz and *D*_0_ varies depending on temperature *T* as Δ*T* = Δ*D*/*α*, where α = −74 kHz/K. (B) Fluorescence image of ND-NG-ICG in a living cell after 4 h of incubation using a ND-NG-ICG concentration of 10 μg/mL (the dashed line is the cell border). (C) Representative ODMR spectra of ND-NG-ICG that were fitted with a double Lorentzian under near-infrared (NIR) irradiation (810 nm lamp; 0.35 W/cm^2^) at 0 and 3 min after 4 h of incubation using a ND-NG-ICG concentration of 10 μg/mL. (D) Thermal profiles of ND-NG and ND-NG-ICG at concentrations of 10, 50, 100, and 200 μg/mL under NIR irradiation (810 nm lamp; 0.35 W/cm^2^). (E) Change in intracellular temperature measured by ODMR for NG-NG-ICG and ND-NG over 420 s under NIR irradiation (810 nm lamp; 0.35 W/cm^2^) after 4 h of incubation using a ND-NG-ICG concentration of 10 μg/mL.

To measure the intracellular changes in the local temperature at the nanoscale under irradiation, HeLa cells were cultured on an autoclaved glass coverslip, which was placed in a 12-well cell culture plate and incubated overnight. On the next day, fresh medium containing 10 or 100 μg/mL of ND-NG or ND-NG-ICG was added. After 4 h of incubation and three washings, cells were placed in a home-built confocal fluorescence microscope combined with an optically detected magnetic resonance (ODMR) spectrometer for measurement (Figure 3 and Figures S5, S6, and S8). The zero-field splitting *D*_0_ of the NV^–^ center in ND is temperature-dependent. Therefore, measurement of the zero-field splitting via ODMR provides a robust readout of the local temperature at the nanoscale. As shown in Figure 3B, the NDs are very bright in comparison to the autofluorescence of the cell, and the spots in cells were chosen for ODMR. The ODMR spectra of ND-NG-ICG under NIR irradiation were recorded and fitted with a double Lorentzian (Figure 3C). After 3 min of continuous irradiation, the ODMR spectrum shifted to low frequency, indicating a change in temperature. However, the ND-NG sample (Figure S7) revealed no significant shift in the ODMR spectra under NIR irradiation. To readout and monitor the intracellular changes in temperature, ODMR spectra were recorded up to 7 min continuously and the data were processed every 60 s. The ODMR spectra were fitted with a double-Lorentzian function, and the changes in temperature were extracted according to the equation
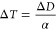
where Δ*D* is the shift in the transition frequency and α = d*D*/d*T* = −74 kHz/K is the temperature susceptibility.^34^ In addition, the irradiation time over the change in temperature was fitted (Figure 3E). We found that the temperature increased sharply by more than 30 °C and was saturated after approximately 250 s of irradiation for the ND-NG-ICG samples, whereas there was no significant change in temperature in the ND-NG samples under irradiation. The result was independent of the concentration, and similar local temperature increases were obtained for ND-NG-ICG concentrations of 10 and 100 μg/mL (Figure 3E and Figure S8).

The operating principle of thermometry using NV^–^ centers relies on the accurate measurement of the transition frequency, which can be optically detected with high spatial resolution. For NV-based thermometry, the temperature sensitivity can be calculated by the equation
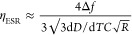
where d*D*/d*T* is the NV temperature susceptibility, Δ*f* is the approximate ODMR line width, *C* is the ODMR contrast, and *R* is the average photon counts. Applying d*D*/d*T* = 74 kHz/K, *C* ≈ 0.05, Δ*f* = 5 MHz, and *R* = 3 M cts/s resulted in a calculated sensitivity of η = 600 mK/√Hz. Theoretically, a single NV^–^ center can potentially exhibit a sensitivity of higher than 1 mK/√Hz.

To increase the sensitivity, NDs without impurities, for example, isotopic ^13^C or ^14^N, would be necessary as well as NDs with a reduced number of NV centers.^29^ In addition, instead of recording the full ODMR spectra, a four-point method was reported,^29^ which measured only the fluorescence at the steepest point of the ODMR spectrum. However, in this way, important information in the ODMR spectrum could be lost, such as the effect of external magnetic ions from frequency splitting and microwave intensity from the ODMR contrast. Therefore, knowledge of the ODMR line shape is required before temperature measurements.^35^ Furthermore, the process can be improved by replacing the NIR LED lamp with a NIR laser in combination with a self-calibrating real-time particle tracking algorithm.^36^

## Temperature Measurements at the Macroscopic Level

To correlate the changes in temperature from the nanoscale to a macroscopic level, the photothermal effect of ND-NG-ICG was studied using a thermocouple in aqueous solution. The photothermal effect of ND-NG-ICG was measured under the same conditions (810 nm lamp, 0.35 W/cm^2^) that were applied during ODMR spectroscopy. First, we evaluated the change in temperature of ND-NG in the absence of the ICG photothermal probe at different concentrations (Figure 3D) in aqueous solvent. A concentration-independent temperature increase of less than 2 °C was detected, which reached a saturation after 5 min of irradiation, indicating that ND-NG did not possess photothermal activity. In contrast, free ICG and ND-NG-ICG showed similar and pronounced concentration-dependent temperature increases. After 5 min of irradiation of free ICG, temperature changes of 11 °C (10 μg/mL of ICG) up to 36 °C (200 μg/mL of ICG) were detected. Irradiation of ND-NG-ICG for 5 min induced a temperature increase of 2 °C (10 μg/mL of ND-NG-ICG with a loaded ICG content of 2.44 μg/mL) up to 18 °C (200 μg/mL of ND-NG-ICG with a loaded ICG content of 48.77 μg/mL). These results indicate that temperature changes measured at the macroscopic level in the bulk solvent are much lower than the temperature changes at the nanoscale, probably due to heat dissipation.

## ND-NG-ICG Serves as a Temperature-Reporting Heating Source Displaying a Pronounced Photothermal Effect in Live Cells

**Figure 4:**
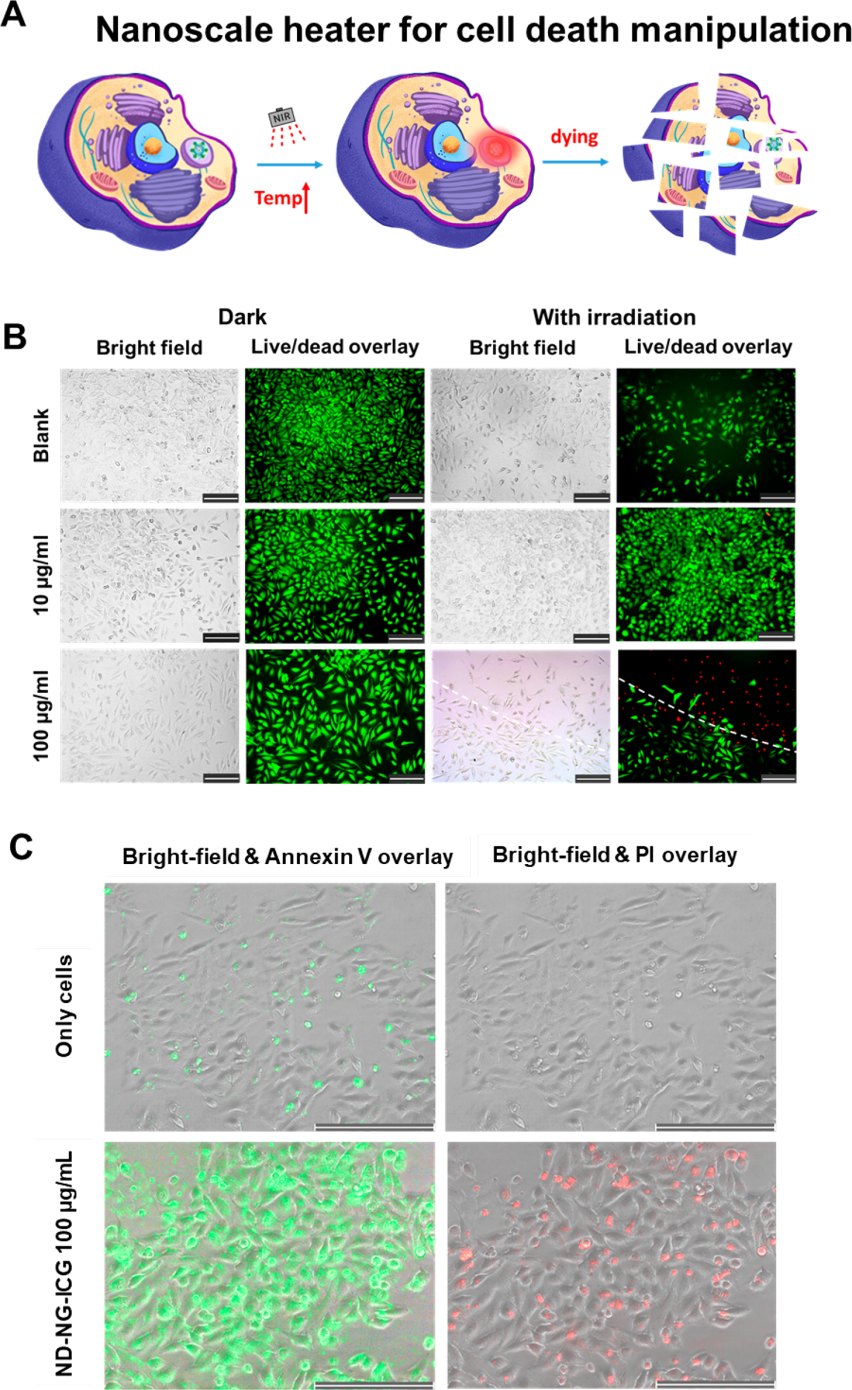
(A) Sketch of a nanoscale heater for cell death manipulation. (B) Live/dead staining of HeLa cells incubated at different concentrations of ND-NG-ICG after 20 min irradiation using a near-infrared (NIR) LED lamp (810 nm lamp; 0.35 W/cm^2^; scale bar 200 μm). Green and red represent live and dead cells, respectively. (C) Early apoptosis detection with Annexin V staining. HeLa cells were incubated with ND-NG-ICG (100 μg/mL) and irradiated for 20 min using a near-infrared (NIR) LED lamp (810 nm lamp; 0.35 W/cm^2^; scale bar 200 μm). Green and red represent apoptotic and dead cells, respectively.

To understand the nanoscale photothermal effects on cells, LED light with a wavelength of 810 nm was focused onto the sample area through the objective of a microscope for 20 min. The light power density was set to 0.35 W/cm^2^, which was the same value we used to measure the local temperature by ODMR spectroscopy. After 20 min of irradiation, cells were further incubated for 4 h and analyzed by live/dead staining (Figure 4A). As shown in Figure 4B, without irradiation at 810 nm, cells could proliferate well and show a normal cell morphology, and almost all cells remained viable after incubation with 10 or 100 μg/mL of ND-NG-ICG. After irradiation at 810 nm, the cell viability was not impaired for low concentrations of ND-NG-ICG (10 μg/mL) and ND-NG (Figure S9). However, most of the cells treated with a higher concentration of ND-NG-ICG (100 μg/mL) were found to be dead in the live/dead imaging strictly within the irradiated area. The initiation of cell death was further proven by early apoptosis experiments (Figure 4C), which showed the same effect.

On the basis of the ODMR measurements, we recorded the local temperature change (Δ*T*) inside cells. Remarkably, temperature changes of not less than 30 °C after only 250 s of irradiation at 810 nm for a low concentration of ND-NG-ICG (10 μg/mL) and a high concentration of ND-NG-ICG (100 μg/mL) were detected. Surprisingly, the local temperature increase is independent of the nanoparticle concentrations, because both 10 and 100 μg/mL revealed a similar increase in local temperature after heating (Figure 3E and Figure S8). However, no cell death was observed for a low concentration of ND-NG-ICG (10 μg/mL) under irradiation at 810 nm for 20 min. We propose that cells could compensate for a pronounced local temperature increase if only a few heating sources of a low concentration of ND-NG-ICG are present. In this way, our results are in line with other reports: i.e., on mitochondria, which can tolerate comparatively high temperatures without affecting cell viability.^10^ In contrast, the presence of many local ND-NG-ICG heating sources (100 μg/mL), which produced heat over a larger volume in the cell, effectively induced cell death. The confocal microscopy images for 10 and 100 μg/mL of ND-NG-ICG in HeLa cells also revealed that the number of ND-NG-ICGs in the cells increased if the cells were incubated with higher concentrations of ND-NG-ICG (100 μg/mL; Figure S10). However, NDs possess different numbers and types of NV centers, and thus each nanopaticle could emit different amounts of photons. Therefore, quantifying the number of NDs from the fluorescence signal is not possible.

## Conclusion

In summary, we have prepared a nanodiamond-based local heating and nanoscale temperature self-reporting photothermal agent with pronounced bioactivity. This nanoscale sensor was able to monitor the nanoscale temperature increase after heating due to the photothermal effects *in situ* and in living cells. The ODMR results showed an increase in the local temperature inside endosomes of not less than 30 °C within 250 s of irradiation using a concentrations of ND-NG-ICG of 10 and 100 μg/mL. In this case, cells could tolerate a high local temperature increase if the concentration of the photothermal agent is low. These data support that the intracellular temperature can be inhomogeneous and can even differ by 30 °C without affecting cell viability. Our study investigated the nanoscale local temperature change induced by a photothermal agent. This work helps to gain a deeper understanding of the photothermal effect at the nanoscale and paves the way for further studies on the photothermal effect in different intracellular environments and its effect on cell viability. Furthermore, it has been recently reported that local temperature change can influence the cell division time.^36^ An ND-based nanothermometer could represent a valuable tool to investigate temperature-driven biological processes in more detail at the intracellular level or even at the subcellular level.

## Supporting Information Available

Materials and methods, ICG loading calculation curve, concentration ICG release profile *in vitro*, emission spectra of NDs and emission spectra and absorption spectra of ICG, thermal profiles of ICG at different concentrations, schematic of the home-built confocal fluorescence microscope with optically detected magnetic resonance (ODMR) spectroscopy, schematic of the experiment setup for intracellular temperature-sensing measurements, fluorescence image and ODMR spectra of ND-NG in a living cell, live/dead staining of HeLa cells incubated with different concentrations of ND-NG after 20 min of irradiation, confocal microscopy images for 10 and 100 μg/mL ND-NG-ICG in HeLa cells after 4 h of incubation, emission spectrum of ND-NG-ICG in HeLa cells (PDF)
