# Supplementary material for: Nanodiamond Theranostic for Light-Controlled Intracellular Heating and Nanoscale Temperature Sensing
Source: Nano Lett. 2021 Apr 21;21(9):3780–8. doi: 10.1021/acs.nanolett.1c00043 (PMC8289278; doi:10.1021/acs.nanolett.1c00043)
Supplement: Supplementary file 1 — nl1c00043_si_001.pdf [file nl1c00043_si_001.pdf]

Supplementary information

Nanodiamond Theranostic for Light-Controlled Intracellular Heating and Nanoscale Temperature Sensing

Yingke Wu,[†,‡] Md Noor A Alam,[†,‡,§] Priyadharshini Balasubramanian,[ƻ] Anna Ermakova,[‡,||] Stephan Fischer,[ͳ] Holger Barth,[ͳ] Manfred Wagner,[‡] Marco Raabe,[,‡,§] Fedor Jelezko,[*,ƻ] and Tanja Weil,[*,‡,§]*

‡Max Planck Institute for Polymer Research, Ackermannweg 10, 55128 Mainz, Germany

§Institute of Inorganic Chemistry I, Ulm University, Albert-Einstein-Allee 11, 89081 Ulm, Germany

ƻInstitute for Quantum Optics, Ulm University, Albert-Einstein-Allee 11, 89081 Ulm, Germany

||Institute for Physics, Johannes Gutenberg University Mainz, Staudingerweg 7, 55128 Mainz, Germany

ͳInstitute of Pharmacology and Toxicology, University of Ulm Medical Center, 89081 Ulm, Germany

*E-mail: weil@mpip-mainz.mpg.de, raabe@mpip-mainz.mpg.de
fedor.jelezko@uni-ulm.de

Materials: Nanodiamonds with 35 nm average diameter were purchased from FND Biotech (Taiwan), 4-arm PEG-SCM (MW: 10 kDa, Creative PEGWorks), branched polyethylenimine (MW: 25 kDa by LS), polyvinylpyrrolidone (MW: 10 kDa), (3-carboxypropyl)triphenyl-phosphonium bromide, *N*-hydroxysuccinimide (NHS), 9,10-anthracenediyl-bi(methylene)-dimalonic acid, fluorescamine and ethylenediamine, indocyanine green were purchased from Sigma-Aldrich, Dulbecco's Modified Eagle's Medium (DMEM, 1x), Dulbecco's phosphate-buffered saline (DPBS, 1x), fetal bovine serum (FBS), Penicillin Streptomycin (Pen Strep) were purchased from gibco. All solvents and chemicals were purchased from commercial sources and were used without further purification.

Methods

Preparation of ND-NG

Firstly, 100 µL ND (2 mg/mL) stock solution was dispersed in 300 µL water. Afterwards, 100 µL PVP (20 mg/mL), 200 µL PEI (5 mg/mL), and 300 µL PBS buffer (10 mM, pH 7.4) were added. After 5 minutes sonication, 2 mg of 4-arm PEG-SCM was added to cross-link the PEI on the surface of ND. The final volume was adjusted to 1 mL with MilliQ water. After sonication for 30 minutes and reaction for 90 minutes on a shaker at 800 rpm, the obtained ND-NGs were washed three times by centrifugation at 12,000 rpm for 20 minutes.

Preparation of ND-NG-ICG

100 µL of a 2 mg/mL ND-NG solution was mixed with 400 µL of 0.1 mg/mL ICG and was sonicated 30 seconds, then reacted overnight on a shaker at 800 rpm, the ND-NG-ICG were obtained by centrifugation at 12,000 rpm for 20 minutes to remove the supernatant.

Loading efficiency

A standard curve of ICG absorbance was prepared to calculate the ICG content in ND-NG-ICG as followed: a 20 µL ICG solution with concentration of 62.5 µg/mL, 31.25 µg/mL , 15.63 µg/mL, 7.81 µg/mL, 3.91 µg/mL, 1.95 µg/mL water, and supernatant of ND-NG-ICG were added in separate wells of 384-well low volume well plate. And then absorbance intensity at 789 nm were obtained by a Tecan Spark 20M. The absorbance intensity–concentration curve was drawn and the ICG content in ND-NG-ICG was calculated.

ICG releasing curve

200 µg ND-NG-ICG was added to 500 µL MilliQ water and put on a shaker at 800 rpm, at the setting time points, one of the ND-NG-ICG aliquots was centrifuged at 12,000 rpm for 20 minutes, the absorbance of supernatant at 789 nm was measured by a Tecan Spark 20M, and the released ICG content was calculated.

UV-Vis Absorbance and Fluorescence

20 µL of ICG solution (0.1 mg/mL) were prepared in separate wells of a 384-well low volume microplate. Absorbance scans from 200 to 1000 nm were obtained using a Tecan Spark 20M microplate reader. And the emission spectra of ICG was measured using a 40 µL micro cuvette at

Perkin Elmer Lambda 900 device. It was excited at 600 nm and the emission spectra was collected from 750 nm to 1000 nm. And the spectra were normalised.

Transmission Electron Microscopy (TEM)

One drop of a 0.1 mg/mL ND-NGs solution in MilliQ was placed onto an oxygen treated copper grid. A Jeol 1400 transmissions electron microscope was used to obtain bright field images.

Dynamic Light Scattering (DLS)

500 µL of 0.1 mg/mL solutions of ND, ND-NG, or ND-NG-ICG in MilliQ were transferred into a borosilicate glass cuvette and measured at 25 °C with a 90° angle using a particle sizer (Malvern Zetasizer Nano-S90 (Nano series)). The hydrodynamic diameter distribution was presented as intensity. Zeta potential was measured at 25 °C with a Malvern Zetasizer Nano-S90 (Nano series).

Photothermal profile study for bulk ND-NG, ND-NG-ICG and pure ICG solutions in water

Various concentration of ND-NG, ND-NG-ICG and pure ICG were prepared to measure the photothermal temperature changes. Subsequently, a K-type thermocouple wire and an automated temperature input device (USB-TC01, National Instruments) were employed to record the real-time temperature change of 100 µL of each samples. The process were repeated three times for each concentration. An 810 nm LED lamp (Thor Labs, M810L3) was used to irradiate the samples, light irradiance was 0.35 W/cm^2.

Samples preparation of cell with ND-NG-ICG for Transmission Electron Microscopy (TEM)

Cells were cultured in a 24-well plate pre-placed with carbon coated sapphire discs (d:3mm) with a density of 50,000 cells/mL. After co-incubation with nanoparticles (NPs), sapphire discs were placed between two aluminum plates to create a 'sandwich' and were mounted afterwards into a holder (Engineering Office, M. Wohlwend) and immediately fixated in a Wohlwend HPF Compact 01 high-pressure freezer (Engineering Office, M. Wohlwend) with a pressure of 2100 bar. The frozen samples were then stored in liquid nitrogen.

Frozen sapphire discs were carefully removed from the aluminum 'sandwich' and transferred into 1 mL pre-cooled freeze substitution medium (0.2% (w/v) osmium tetroxide, 0.1% (w/v) uranyl acetate, 5% (v/v) distilled water in acetone) and kept in a freeze substitution unit (AFS2,Leica). Samples were then slowly warmed up to 0 °C over a period of 20 hours in the unit. After being warmed up, the freeze-substituted samples were brought to room temperature, then the substitution medium was removed and the discs were washed three times with acetone at half an hour intervals. Then the discs were infiltrated sequentially in gradient epoxy resin-acetone mixture (1;1, 1:2, and 2:1) for 1 h. Samples were then infiltrated in 100% epoxy resin overnight. Finally, each sample was transferred into a new Eppendorf tube containing fresh epoxy resin for polymerization at 60 °C for 24 h.

Following polymerization, sapphire discs were detached using liquid nitrogen. Resin blocks with the cells imprinted on were trimmed and sectioned into 100nm/80nm sections by a 45° diamond knife (Diatome) in EM UC6 ultramicrotome (Leica).

Confocal Microscopy

Confocal laser scanning microscopy (CLSM) was utilized to observe the cellular uptake of ND-NG-ICG. HeLa cells were seeded in an ibidi 8 well µ-slide (10^5 cells/mL, 200 µL each well) and

incubated overnight. The cells were washed once with Dulbecco's phosphate-buffered saline (DPBS) and 100 µg/mL ND-NG-ICG (mixed with cell culture medium) was added to the cells. After four hour incubation at 37 °C, the cells were washed three times with DPBS to remove the particles which were not taken up, then fresh medium was added. A Leica TCS SP5 confocal microscope system coupled to a 63× water immersion objective was used to obtain the microscopy images and later they were analyzed with ImageJ software. Images were recorded using a 561 nm laser for excitation and 660–700 nm filter for emission.

For the confocal microscopy of HeLa cells with different concentrations of ND-NG-ICG, cells were seeded in an ibidi 8 well µ-slide (120,000 cells/mL, 300 µL each well). After overnight incubation, cells were washed with DPBS, then 10 µg/mL and 100 µg/mL ND-NG-ICG in culture medium were added to the cells (200 µL in each well). Then cells were incubated at 37 °C again for four hours, washed three times with DPBS and confocal microscopy was performed immediately. Images were recorded using a 561 nm laser for excitation and 660–700 nm filter for emission. Same laser power and gain value were used for all the samples during the microscopy.

Cytotoxicity study

HeLa cells were seeded on a white 96 well half-area flat bottom cell culture microplate (Greiner). Seeded cell concentration was 110,000 cell/mL, 50 µL in each well. After incubation at 37 °C overnight and 5% CO_2, various concentrations of ND-NG-ICG and doxorubicin (as negative control) were added to the cells and incubated again for 24 hours. The next day, the cells were washed three times with DPBS and fresh cell culture medium (containing DMEM with 10% FBS, 1% MEM NEAA, and 1% PenStrep) was added. Afterward, freshly prepared CellTiter-Glo

luminescent cell viability assay (Promega) was applied as instructed by the manufacturer's protocol. The luminescence signals were recorded with a Promega GloMax multi detection plate reader. Origin software from OriginLab was used for statistical analysis (one-way ANOVA and Tukey's post-hoc test).

Sample preparation for intracellular temperature measurements

Coverslips of 15 mm diameter were placed in a 12 well cell culture plate to seed HeLa cells. The cell concentration was 10^6 cells/mL, 500 µL in each well. After overnight incubation, 10 µg/mL ND-NG and ND-NG-ICG samples in cell culture medium were added to the cells. Subsequently after four hour incubation at 37 °C, the cells were washed three times with DPBS to remove the excess nanoparticles which were not taken up, and fresh clear DMEM (without phenol red) was added to the cells. The ODMR measurements were performed immediately. As depicted in Figure S6, the cell attached coverslip was put on ODMR sample holder in a sandwich-like formation, where the microwave antenna and the cells were in the middle of two coverslips which was filled with cell culture medium (Figure S6).

The temperature sensing experiments are performed in a homebuilt confocal fluorescence microscope shown in Figure S5. The continuous-wave optically detected magnetic resonance (CW-ODMR) spectrum of isolated nanodiamonds (selected based on fluorescence intensity and the point spread function) is first acquired without IR irradiation for about 60 seconds. To measure the real-time temperature change of ND-NG and ND-NG-ICG due to IR irradiation, the IR lamp and the ODMR measurement are synchronously started. The ODMR data is continuously acquired for 7–10 minutes without any disruption. During post-processing, the ODMR data are binned every

60 seconds and the resulting ODMR spectrum was fitted with a double Lorentzian function. The shift in the peak frequencies of the NVs ODMR spectrum was used to determine the real-time intracellular temperature change.

One of the major challenges of the presented time-resolved temperature sensing experiment is the sudden positional drift of the NDs. The effect was more pronounced for ND-NG-ICG samples due to local heating. This leads to the large discrepancy in some data points of Figure 3E (main text) and Figure S8C. Such positional drift of NDs can be corrected by using real-time particle tracking techniques as demonstrated by Choi et al.[1] The CW ODMR sensing scheme used here has a limited time resolution as each OMDR sweep with 100 frequency points takes about one second. Although the time resolution was sufficient to capture the dynamics of temperature change of ICG due to IR irradiation, this can be considerably reduced by measuring the fluorescence at only the steepest point of the ODMR spectrum (or a four-point method) as demonstrated by Kucsko et al.[2]

Live/dead staining of HeLa cell

HeLa cells were seeded on two ibidi 8 well µ-slide with a cell concentration of 160,000 cells/mL (300 µL in each well) and incubated overnight. Subsequently 10 µg/mL and 100 µg/mL ND-NG and ND-NG-ICG samples were prepared by diluting the stock solution with cell culture medium. 200 µL of each concentration of samples were added to the cells and kept in the incubator for four hours. Then both plates were washed three times with DPBS, and added fresh culture medium. Meanwhile, the 810 nm LED was mounted to a Leica DMi8 microscope using a microscope collimation adapter (Thor Labs) to irradiate one of the treated µ-slides for 20 minutes (0.35 W/cm^2). An incubator was used during the irradiation to keep the cells in 37 °C to simulate human body

temperature. After photo-treating the cells, both µ-slides were kept in an incubator for additional four hours. Afterward, cells were treated with freshly prepared live/dead staining solution. The solution was prepared as stated in the ibidi protocol. Briefly 8 µL fluorescein diacetate (5 mg/mL) and 100 µL of propidium iodide (1 mg/mL) were mixed with DPBS (5 mL). 300 µL of live/dead staining solution was added to each well of the µ-slide and kept in dark for five minutes. Then, the cells were washed with DPBS. Imaging was performed immediately using a Leica DMi8 microscope with a Leica MC170 HD camera system.

Early apoptosis detection with Annexin V staining

HeLa cells were grown in a 10 well CELLview Greiner Bio-One cell culture slide (10^6 cells/mL, 100 µL in each well). Next day, 100 µg/mL ND-NG-ICG in cell culture medium was added to some of the wells (100 µL each). After 4 hours of incubation, cells were washed with DPBS and then treated with 810 nm light same way as earlier mentioned in live/dead staining of the HeLa cells. Then again after 4 hours incubation, eBioscience™ Annexin V-FITC apoptosis detection kit was used to stain the cells simultaneously with propidium iodide (PI). Stock 10x binding buffer from the assay kit was diluted to 1x. For each 200 µL diluted binding buffer, 5 µL of Annexin V-FITC and 5 µL PI were mixed, then 100 µL of staining solution was added to each well and left in dark for 15 minutes at room temperature. After 15 minutes, the staining solution was replaced with 1x binding buffer and immediately the microscopy was performed.

Results

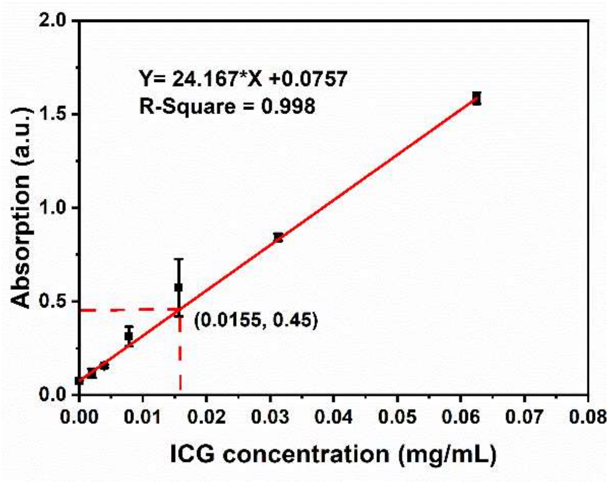

Figure S1. The standard curve of absorbance at 789 nm and ICG concentration.

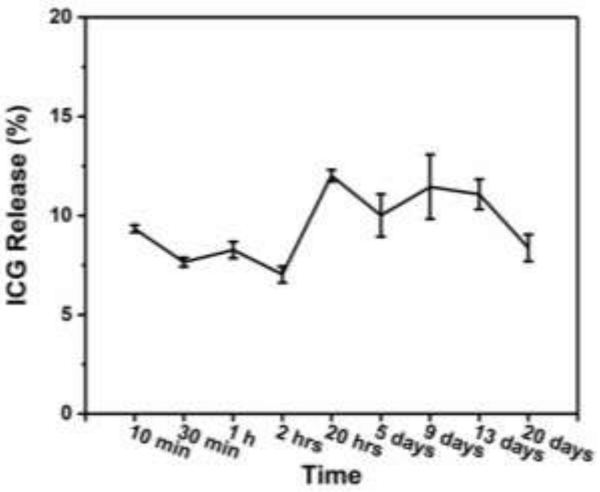

Figure S2. ICG release profile in vitro.

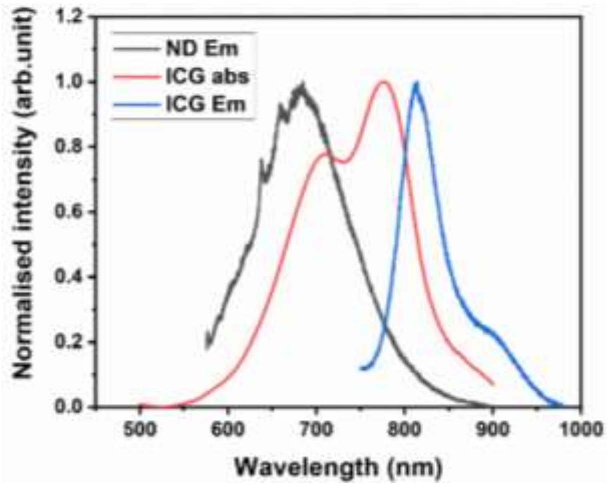

Figure S3. Normalized emission spectra (ex. 532 nm) of ND (black), normalized absorption spectra of ICG (red), and normalized emission spectra (ex. 600 nm) of ICG (blue). The ICG spectra reported in literature.[3, 4]

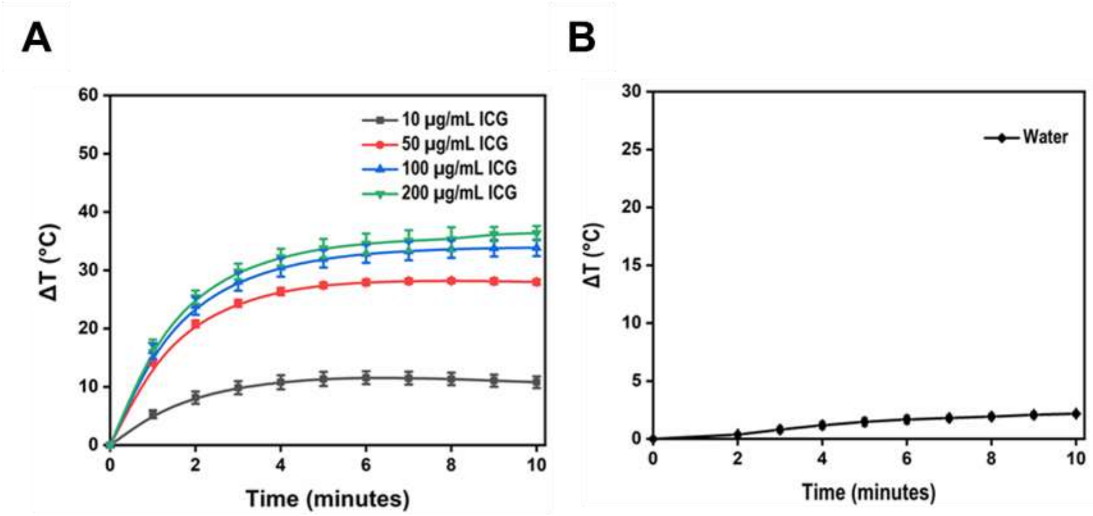

Figure S4 (A) Thermal profile of ICG at a concentration of 10 µg/mL, 50 µg/mL, 100 µg/mL, and 200 µg/mL under near-infrared (NIR) irradiation (810 nm lamp; 0.35 W/cm^2). (B) Thermal profile of water under near-infrared (NIR) irradiation (810 nm lamp; 0.35 W/cm^2).

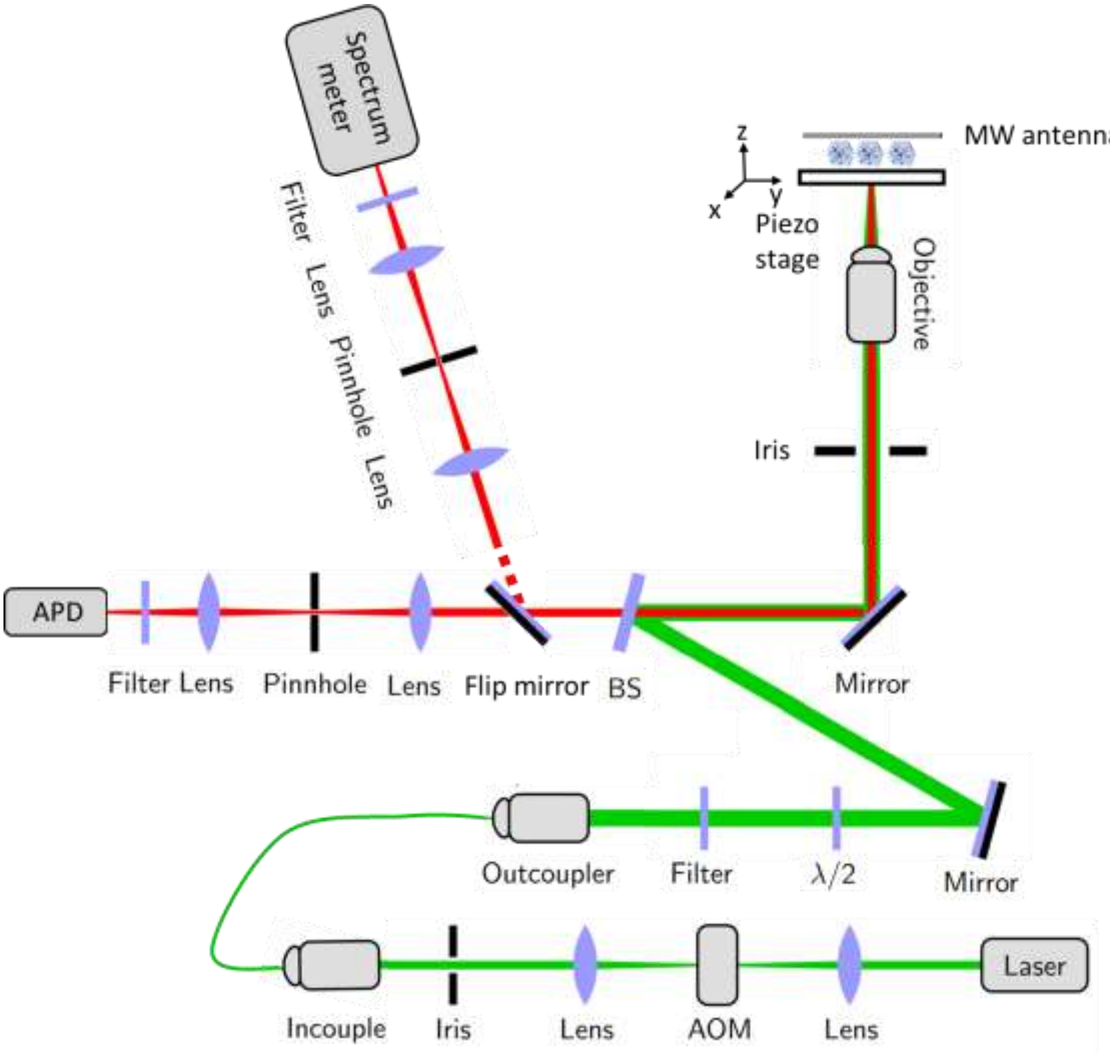

Figure S5. Schematic of home-built confocal fluorescence microscope with optically detected magnetic resonance (ODMR) spectroscopy. Briefly, a 532 nm wavelength laser (Laser Quantum gem 532) is used to excite the NV center, A lens focused the beam coming from the laser source onto a TeO2 acousto-optic modulator (AOM) (Crystal Technology 3200-146), which is used as an optical switch in the pulsed experiments. After the AOM, the laser is coupled into a single-mode fiber, the fiber thereby acted as a mode cleaner, since it couples only the Gaussian TEM00 mode. It was necessary because the clean Gaussian mode produced by the laser can be distorted while passing the AOM. The outcoupled light from the fiber subsequently passed a 530 nm notch filter (Chroma Technology HQ530/30M) to filter out potential fluorescence arising from the fiber, and a λ/2-plate (Thorlabs WPH10M-532), which enables polarization adjustments. Another mirror then lead the beam to a beam-sampler (BS) (ThorlabsBSF20-B), which has the property to be mainly transparent for the NV center's fluorescence, but still reflects some parts of the green light towards an immersion oil objective (Olympus UPLSAPO60XO). It was used to focus the green light down to the diamond sample, as well as to collect the fluorescence light and direct it back towards the beam-sampler. By means of a 3D-piezostage with a scan range of 200×200×25 μm together with an accuracy of 0.5 nm (NPoint NPXY200Z25A), scanning and positioning of the

objective and therefore the focal spot was possible. The emission was adjusted by flipping a mirror to either a spectrometry (Princeton Instrument Acton-SpectraPro SP-2500) or an avalanche photodiode (APD) (Excelitas Technologies SPCMAQRH-15) to get spectra or confocal image.

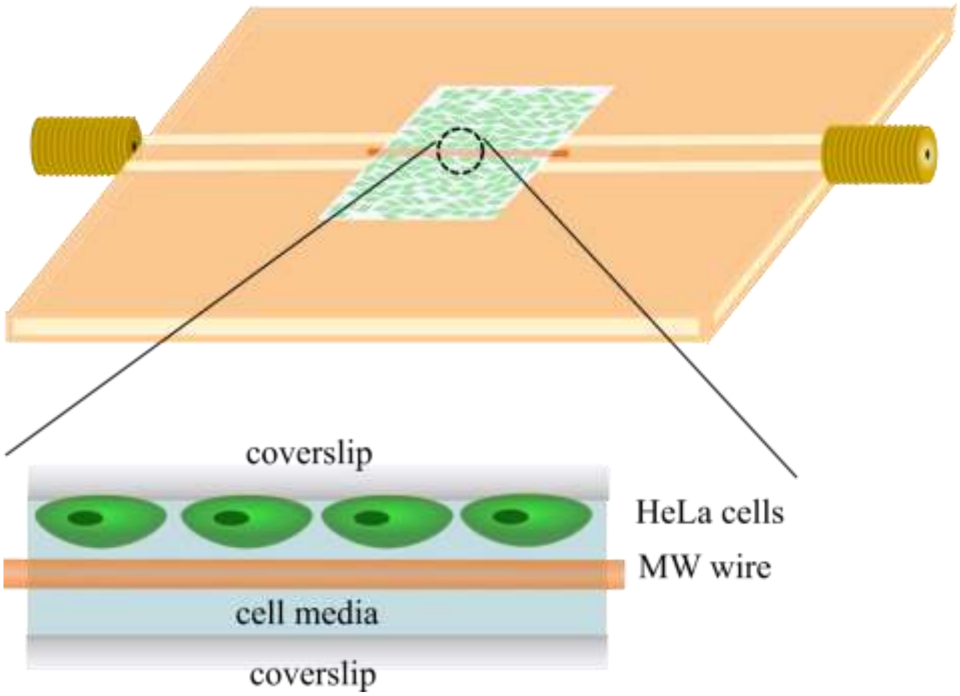

Figure S6. Schematic of the experiment setup for intracellular temperature sensing measurements.

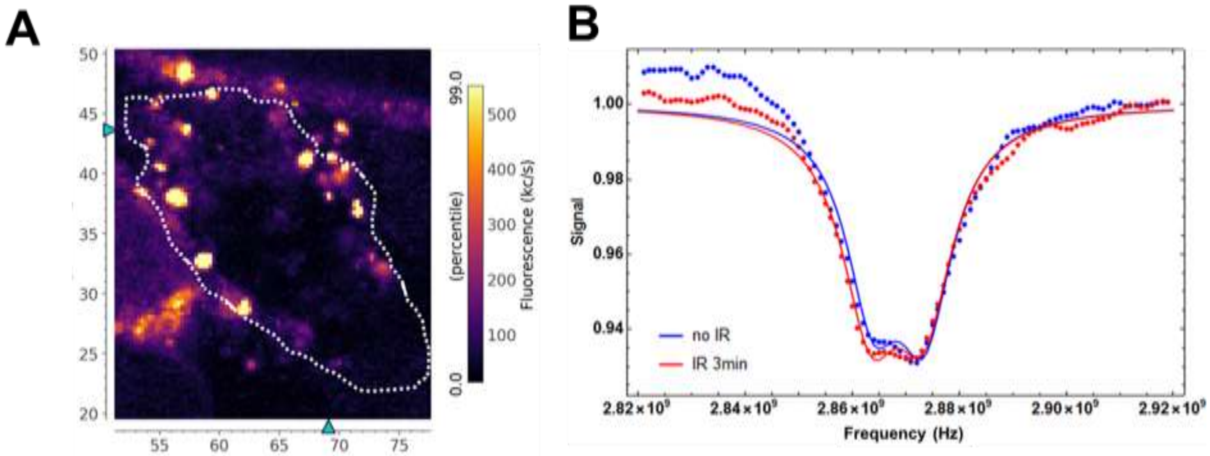

Figure S7. (A) Fluorescence image of ND-NG in a living cell, after 4 hours of incubation using a ND-NG concentration of 10 μg/mL. (B) Representative ODMR spectra of ND-NG at 10 μg/mL which was fitted with a sum of two Lorentzian functions, under near-infrared (NIR) irradiation (810 nm lamp; 0.35 W/cm^2) at the time of 0 min and 3 min.

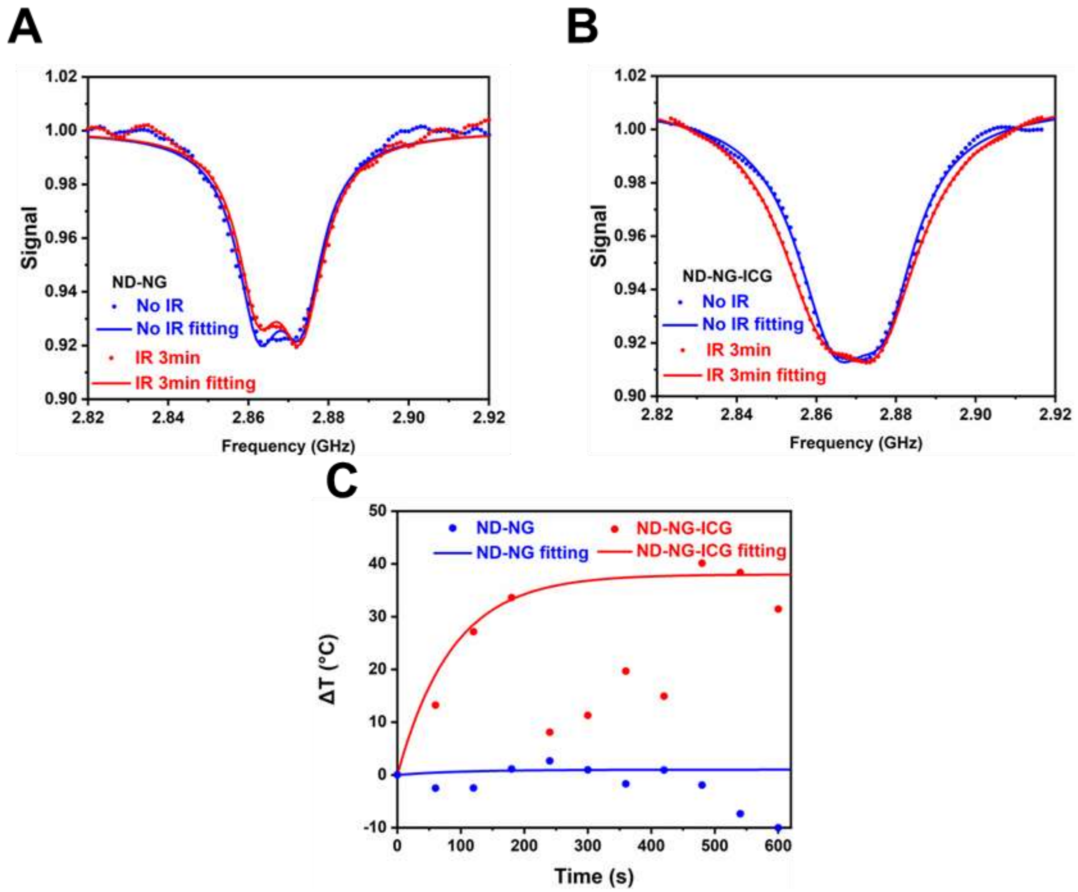

Figure S8. (A) Representative ODMR spectra of ND-NG which was fitted with a double Lorentzian under near-infrared (NIR) irradiation (810 nm lamp; 0.35 W/cm^2) at 0 minute and 3 minutes, after 4 hours of incubation using a ND-NG-ICG concentration of 100 µg/mL. (B) Representative ODMR spectra of ND-NG-ICG which was fitted with a double Lorentzian under near-infrared (NIR) irradiation (810 nm lamp; 0.35 W/cm^2) at 0 minute and 3 minutes, after 4 hours of incubation using a ND-NG-ICG concentration of 100 µg/mL. (C) The change of intracellular temperature measured by ODMR for NG-NG-ICG and ND-NG over 600 seconds under near-infrared (NIR) irradiation (810 nm lamp; 0.35 W/cm^2), after 4 hours of incubation using a ND-NG-ICG concentration of 100 µg/mL.

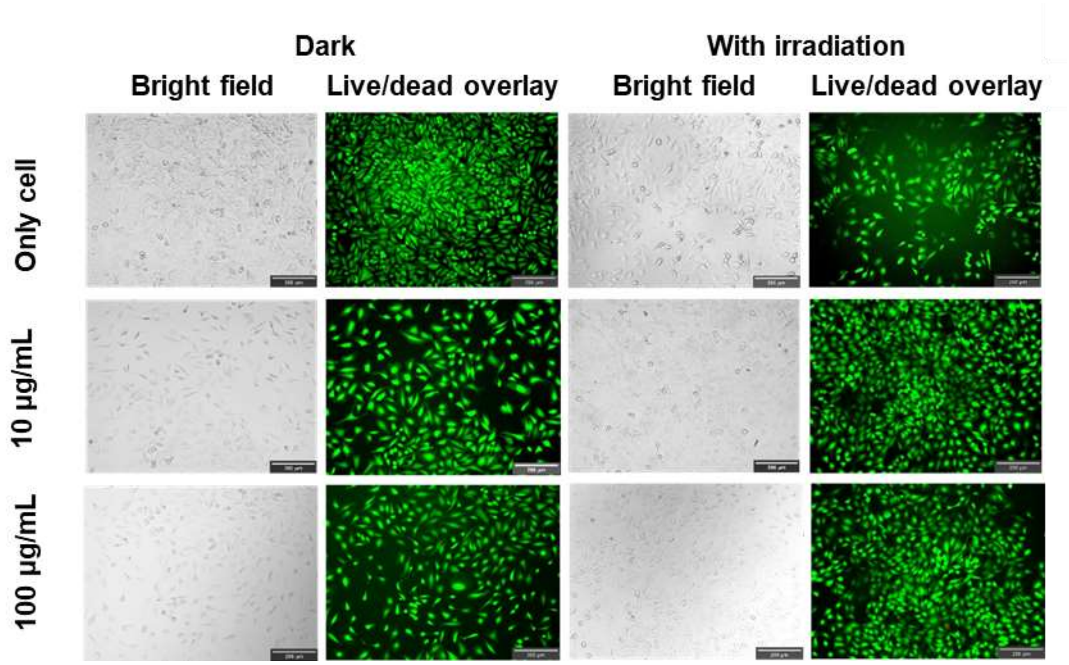

Figure S9. Live/dead staining of HeLa cells incubated with different concentration of ND-NG after 20 minutes irradiation using a near-infrared (NIR) LED lamp (810 nm lamp; 0.35 W/cm^2; scale bar = 200 µm). Green and red color stands for live and dead cells respectively.

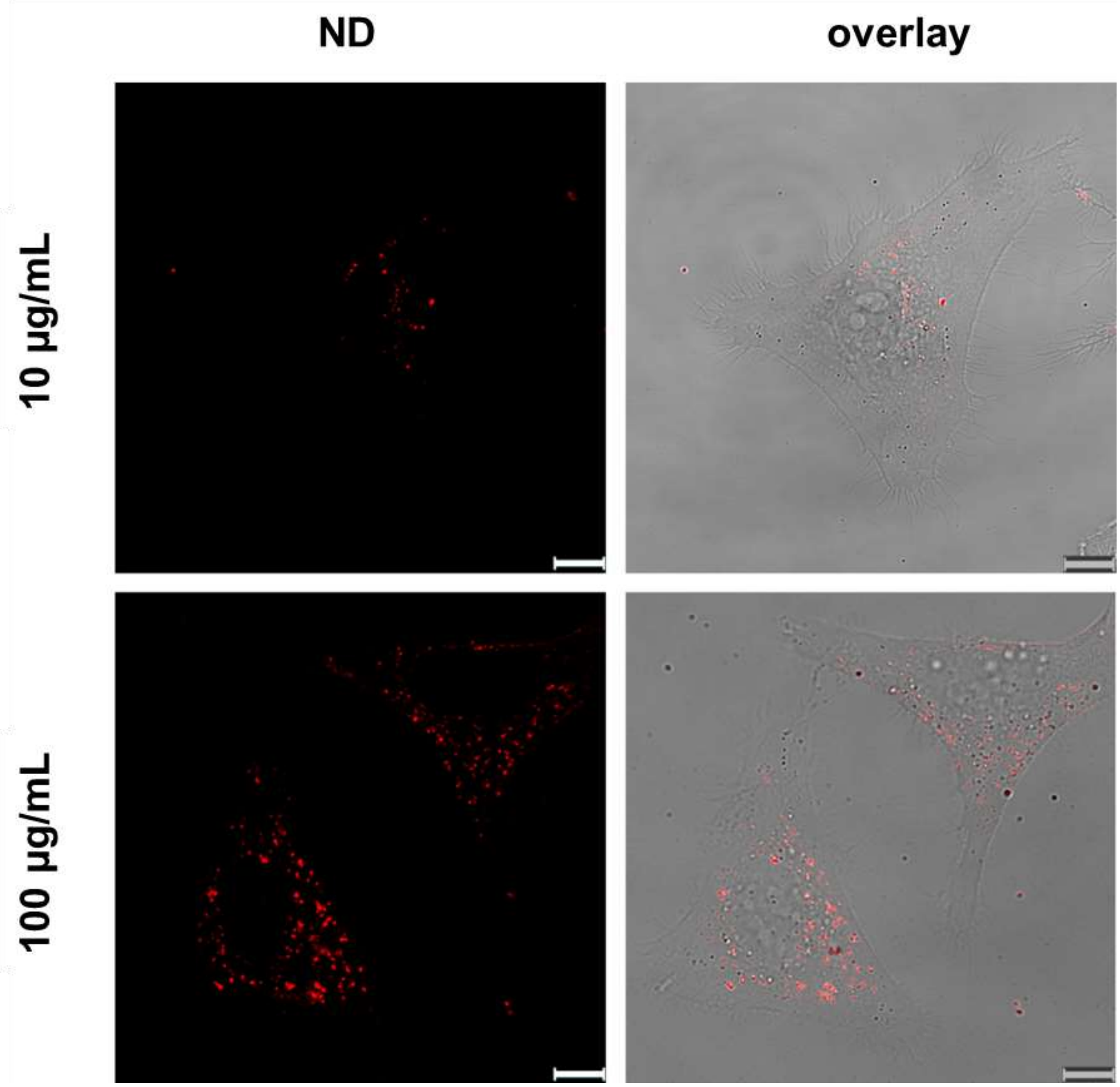

Figure S10. Confocal microscopy images for 10 µg/mL and 100 µg/mL ND-NG-ICG in HeLa cells after 4 hour incubation. Red indicates fluorescence signals from ND. Excitation laser was 561 nm, scale bar 10 µm.

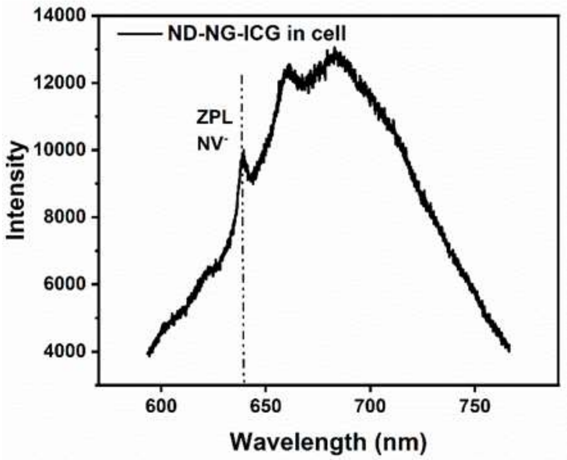

Figure S11. Emission spectrum of ND-NG-ICG in HeLa cells after 4 hours of incubation (Excitation: 532 nm).

References

1. Choi, J.; Zhou, H.; Landig, R.; Wu, H.-Y.; Yu, X.; Von Stetina, S.; Kucsko, G.; Mango, S.; Needleman, D.; Samuel, A. D., Probing and manipulating embryogenesis via nanoscale thermometry and temperature control. *arXiv preprint arXiv:2001.02664* **2020**.
2. Kucsko, G.; Maurer, P. C.; Yao, N. Y.; Kubo, M.; Noh, H. J.; Lo, P. K.; Park, H.; Lukin, M. D., Nanometre-scale thermometry in a living cell. *Nature* **2013,** *500* (7460), 54-58.
3. Sadoqi, M.; Riseborough, P.; Kumar, S., Analytical models for time resolved fluorescence spectroscopy in tissues. *Phys. Med. Biol.* **2001,** *46* (10), 2725.
4. Almarhaby, A.; Lees, J.; Bugby, S.; Alqahtani, M.; Jambi, L.; McKnight, W.; Perkins, A., Characterisation of a near-infrared (NIR) fluorescence imaging systems intended for hybrid gamma-NIR fluorescence image guided surgery. *Journal of Instrumentation* **2019,** *14* (07), P07007.
